# Urinary Excretion of Biomolecules Related to Cell Cycle, Proliferation, and Autophagy in Subjects with Type 2 Diabetes and Chronic Kidney Disease

**DOI:** 10.3390/biomedicines12030487

**Published:** 2024-02-22

**Authors:** Anton I. Korbut, Vyacheslav V. Romanov, Vadim V. Klimontov

**Affiliations:** Laboratory of Endocrinology, Research Institute of Clinical and Experimental Lymphology—Branch of the Institute of Cytology and Genetics, Siberian Branch of Russian Academy of Sciences (RICEL—Branch of IC&G SB RAS), 630060 Novosibirsk, Russia

**Keywords:** type 2 diabetes, chronic kidney disease, albuminuria, glomerular filtration rate, PI3K/AKT/mTOR pathway, PTEN, SIRT1, Klotho, fibroblast growth factor 21, connective tissue growth factor

## Abstract

Dysregulation of cell cycle, proliferation, and autophagy plays a pivotal role in diabetic kidney disease. In this study, we assessed urinary excretion of molecular regulators of these processes that mediate their effects via the PI3K/AKT/mTOR pathway in subjects with long-term type 2 diabetes (T2D) and different patterns of chronic kidney disease (CKD). We included 140 patients with T2D and 20 non-diabetic individuals in a cross-sectional study. Urinary PTEN, Beclin-1, sirtuin 1 (SIRT1), Klotho, fibroblast growth factor 21 (FGF21), and connective tissue growth factor (CTGF) were assessed using ELISA. Patients with T2D, when compared to control, demonstrated increased excretion of PTEN, Beclin-1, SIRT1, FGF21, CTGF, and decreased urinary Klotho (all *p* < 0.05). In the diabetic group, PTEN, FGF21, and CTGF were significantly higher in patients with declined renal function, while Klotho was lower in those with elevated albuminuria. FGF21 and PTEN correlated inversely with the estimated glomerular filtration rate. There was a negative correlation between Klotho and urinary albumin-to-creatinine ratio. In multivariate models, Klotho and PTEN were associated with albuminuric CKD independently. The results provide further support for the role of PTEN, BECN1, FGF21, Klotho, and CTGF in development albuminuric and non-albuminuric CKD in diabetes.

## 1. Introduction

The prevalence of chronic kidney disease (CKD), as well as type 2 diabetes (T2D), has been rising globally [1,2,3]. Moreover, CKD due to T2D and hypertension accounted for the largest disease burden, with 85% incident cases [4]. Recent studies have demonstrated the increasing proportion of declined renal function without preceding or accompanying albuminuria in patients with diabetes and CKD [5,6,7]. Albuminuric and non-albuminuric CKD patterns demonstrate some differences in their risk factors, clinical course, and biomarker profiles [7,8,9], which suggests the specificity of the underlying molecular mechanisms.

Diabetic kidney disease is considered to be associated with the proliferation of mesangial cells, activation of apoptosis in podocytes and tubular epithelial cells, and suppression of glomerular and tubular autophagy [10,11]. These cellular events are mediated by dysregulation of intracellular signaling pathways induced by hyperglycemia and excessive glucose variability [12,13].

The phosphatidylinositol 3-kinases/protein kinase B/mammalian target of the rapamycin (PI3K/AKT/mTOR) signaling pathway plays an essential role in the implementation of the high glucose effect in diabetic kidney disease and renal fibrosis [14,15,16]. The PI3K/AKT/mTOR is a crucial pathways in the regulation of the cell cycle and proliferation [17]. Dysregulation of the PI3K/AKT/mTOR has been demonstrated in obesity, metabolic syndrome and T2D [18,19,20]. PI3Ks are a family of lipid kinases that phosphorylate intracellular inositol lipids to regulate signaling and intracellular vesicular traffic. The class III PI3Ks are regulators of membrane traffic along the endocytic route in endosomal recycling and autophagy [21,22]. Phosphatase and tensin homolog (PTEN) is considered to act as a key suppressor of the PI3K/AKT/mTOR signaling pathway [23,24]. PTEN dephosphorylates phosphatidylinositol-3,4,5-phosphate (PIP3), a critical second messenger of the effects of growth factors and insulin [25]. Klotho protein is another regulator of the PI3K/AKT/mTOR signaling pathway. Klotho decreases expression of Beclin-1 (BECN1) and inhibits the BECN1-Bcl-2 interaction that mediate autophagy and apoptosis [26]. BECN1 is a component of the Class III PI3K complex, which initiates the assembly of autophagosomes from pre-autophagic structures [27]. The PI3K/AKT/mTOR pathway and Klotho were found to regulate the effects of fibrogenic factors. Specifically, Klotho is required for the high-affinity binding of fibroblast growth factor (FGF) receptors with FGF19, FGF21, and FGF23 [28]. Consequently, FGF mediates the induction of the PI3K/AKT/mTOR signaling pathway [29,30,31]. The expression and effects of connective tissue growth factor (CTGF), a fibrotic mediator, also depend upon the PI3K/AKT/mTOR [32,33]. Silent information regulator 1 (SIRT1) is another cell cycle and proliferation regulator that activates the PI3K/AKT/mTOR [34,35].

In this study, we tested hypothesis that molecules regulating cell cycle, proliferation, autophagy and other biological processes in the kidney through the PI3K/AKT/mTOR signaling pathway can be perspective biomarkers of albuminuric and/or non-albuminuric CKD in T2D. Accordingly, we studied the urinary excretion of PTEN, BECN1, SIRT1, Klotho, FGF21, and CTGF in patients with T2D and albuminuric or non-albuminuric CKD.

## 2. Materials and Methods

### 2.1. Design

We performed an observational single-center cross-sectional study. One hundred and forty adult subjects with T2D, 70 men and 70 women, were selected from the institutional database. Eligible patients were required to have at least 10 years of diabetes duration from diagnosis. Verified non-diabetic CKD, end-stage renal disease, acute kidney injury, cancer, and chronic inflammatory disease in medical history were applied as the exclusion criteria. We also did not include individuals with body mass index (BMI) ≥ 40 kg/m^2^ or <18.5 kg/m^2^, and those with major amputations or bariatric surgery in anamnesis.

According to the estimated glomerular filtration rate (eGFR) and urinary albumin-to-creatinine ratio (UACR), four groups of patients were formed. Patients with eGFR ≥ 60 mL/min × 1.73 m^2^ and UACR < 3.0 mg/mmol were included in the normal renal function and normal albuminuria (NF/NA) group. Those with eGFR < 60 mL/min × 1.73 m^2^ and UACR < 3.0 mg/mmol formed the declined renal function and normal albuminuria (DF/NA) group. Patients with eGFR ≥ 60 mL/min × 1.73 m^2^ and UACR ≥ 3.0 mg/mmol were assigned into the normal renal function and elevated albuminuria (NF/EA) group. Finally, individuals with eGFR < 60 mL/min × 1.73 m^2^ and UACR ≥ 3.0 mg/mmol formed the declined renal function and elevated albuminuria (DF/NA) group.

In total, 20 subjects without diabetes, obesity and CKD, 10 men and 10 women, were enrolled into the control group.

### 2.2. Methods

The levels of hemoglobin A1c (HbA1c), serum and urinary creatinine, and urinary albumin were assessed with AU680 Chemistry Analyzer (Beckman Coulter, Brea, CA, USA). eGFR was calculated according to CKD-EPI formula (2009).

The morning urine samples were stored at −20 °C without melt-freeze cycles for following research. The concentrations of PTEN, BECN1, SIRT1, Klotho, FGF21, and CTGF were assessed using ELISA with commercially available kits: SEF822Hu for PTEN, SEJ557Hu for BECN1, SEE918Hu for SIRT1, SEH757Hu for Klotho, SEC918Hu for FGF21, and SEA010Hu for CTGF (Cloud Clone Corp., Wuhan, China). The results were adjusted to the urinary creatinine concentrations.

### 2.3. Statistical Analysis

The continuous variables were tested for normal distribution with a Shapiro–Wilk test (SPSS Statistics, IBM, Armonk, NY, USA). As most of the studied parameters were not distributed normally, the data are presented as medians and interquartile ranges (IQRs). The statistical significance of differences between groups were tested with Mann–Whitney U-tests for comparison of two groups. We used Kruskal–Wallis H-test for multiple group comparisons. The χ2 test was applied for categorical data (SPSS Statistics, IBM, Armonk, NY, USA). The differences were noted as significant with *p*-value below 0.05.

The associations between continuous parameters were assessed with a Spearman correlation analysis (Statistica 13.0, Dell, Round Rock, TX, USA). The associations of urinary excretion of PTEN, BECN1, SIRT1, FGF21, Klotho, and CTGF with declined renal function and elevated albuminuria were tested in receiver operating characteristic (ROC) analysis (SPSS Statistics, IBM, Armonk, NY, USA) and in multiple logistic regression models (Statistica 13.0, Dell, Round Rock, TX, USA).

## 3. Results

### 3.1. Clinical Characteristics of the Study Participants

Clinical characteristics of patients are presented in Table 1. We found some differences in age, diabetes duration, and HbA1c levels between the groups. Patients in DRF/NA group were older; those in DRF/EA group had the longest diabetes duration. The highest levels of HbA1c were observed in NRF/EA group.

All patients received antihyperglycemic medications, including metformin (*n* = 105), sulfonylurea (*n* = 55), dipeptidyl peptidase-4 (DPP4) inhibitors (*n* = 18), glucagon-like peptide-1 (GPL-1) analogues (*n* = 2), sodium/glucose cotransporter 2 (SGLT2) inhibitors (*n* = 35) and insulin (*n* = 96). Most patients (*n* = 115) were treated with renin-angiotensin system blockers. Compared to other groups, lower proportion of DF/EA patients received metformin, while calcium channel blockers were used more frequently in this group (Table 2). There were no significant differences in other treatment modalities between diabetic groups.

The median age of control subjects was 62.5 years (IQR 59.5–67.4 years), and median body mass index (BMI) was 25.7 kg/m^2^ (IQR 24.7–28.6 kg/m^2^). There were no significant differences between control and diabetic subjects in these parameters.

### 3.2. Urinary Excretion of PTEN, BECN1, SIRT1, Klotho, FGF21, and CTGF

**Figure 1:**
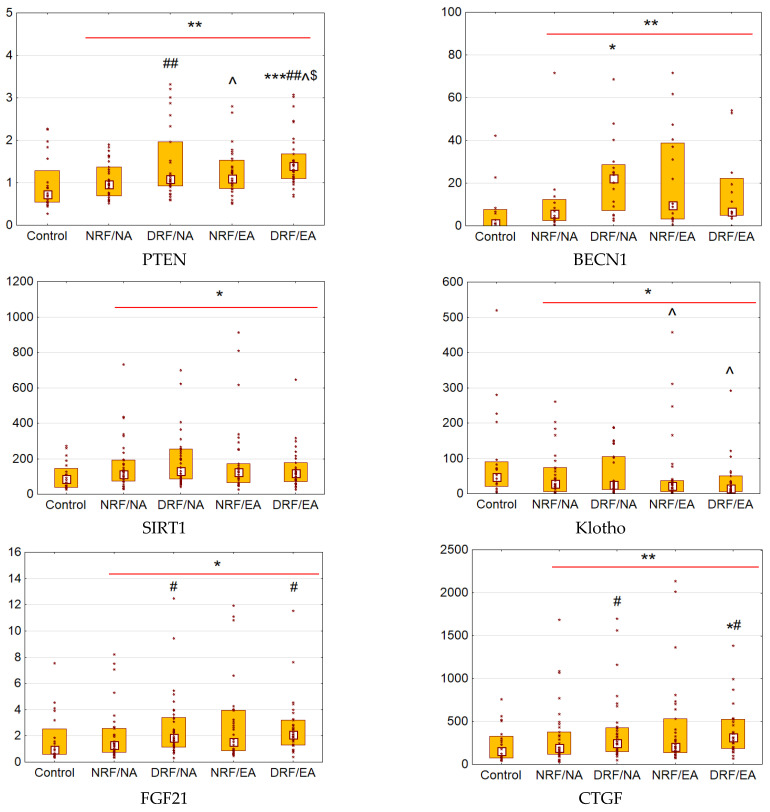
Urinary excretion of phosphatase and tensin homolog (PTEN, ng/mmol), Beclin-1 (BECN1, ng/mmol), sirtuin 1 (SIRT1, ng/mmol), Klotho (pg/mmol), fibroblast growth factor 21 (FGF21, ng/mmol), and connective tissue growth factor (CTGF, ng/mmol) in T2D patients with different CKD status. Data are presented as a bar graphs (median, lower, and upper quartile) and individual data set (dots); * *p* < 0.05, ** *p* < 0.01, *** *p* < 0.001 vs. control; # *p* < 0.05, ## *p* < 0.01 vs. participants with T2D and eGFR ≥ 60 mL/min × 1.73 m^2^; ^ *p* < 0.05 vs. participants with T2D and UACR < 3.0 mg/mmol; $ *p* < 0.05 vs. NRF/NA group. NRF/NA, normal renal function/normal albuminuria group; DRF/NA, declined renal function/normal albuminuria group; NRF/EA, normal renal function/elevated albuminuria group; DRF/EA, declined renal function/elevated albuminuria group.

Patients with T2D demonstrated increased urinary excretion of PTEN, SIRT1, FGF21, and CTGF compared to control (*p* = 0.003 for PTEN, *p* = 0.02 for SIRT1, and *p* = 0.03 for CTGF and FGF21; Figure 1). The most prominent elevation was found in excretion of BECN1 (*p* = 0.004). Meanwhile, Klotho excretion was decreased (*p* = 0.045).

Patients with T2D and eGFR < 60 mL/min × 1.73 m^2^ had a higher excretion of PTEN compared to those with eGFR ≥ 60 mL/min × 1.73 m^2^ (*p* = 0.004). Similarly, patients with UACR ≥ 3.0 mg/mmol showed higher PTEN compared to normo-albuminuric subjects (*p* = 0.03). Patients in DRF/EA group were characterized by significantly elevated urinary PTEN (*p* = 0.001 vs. control and *p* = 0.007 vs. NRF/NA). Otherwise, DRF/NA group demonstrated increased BECN1 when compared to control (*p* = 0.01).

Subjects with T2D and eGFR < 60 mL/min × 1.73 m^2^, when compared to those with eGFR ≥ 60 mL/min × 1.73 m^2^, demonstrated increased urinary excretion of FGF21 (*p* = 0.04) and CTGF (*p* = 0.04). Patients with UACR ≥ 3.0 mg/mmol had lower excretion of Klotho compared to normo-albuminuric ones (*p* = 0.047).

Urinary PTEN and FGF21 demonstrated weak negative correlations with eGFR (r = −0.28, *p* < 0.001, and r = −0.22, *p* = 0.008, respectively), while Klotho correlated negatively with UACR (r = −0.20, *p* = 0.02). Other molecules demonstrated no significant correlations with eGFR and UACR.

Women with T2D had higher excretion of BECN1 and FGF21 than men (BECN1: median 6.2, IQR 3.8–11.4 ng/mmol for men, median 20, IQR 4.3–47.7 ng/mmol for women, *p* = 0.03; FGF21: median 1.3, IQR 0.9–2.6 ng/mmol for men, median 2.2, IQR 1.1–3.5 ng/mmol for women, *p* = 0.01). Oppositely, Klotho was lower in men (median 17.8, IQR 6.7–37.2 pg/mmol for men, median 27.9, IQR 12.2–105.6 pg/mmol for women, *p* = 0.02). PTEN, BECN1, and FGF21 correlated positively with age (PTEN: r = 0.28, *p* < 0.001; BECN1: r = 0.35, *p* = 0.004; FGF21: r = 0.21, *p* = 0.01). In addition, CTGF and FGF21 correlated positively with duration of diabetes (CTGF: r = 0.20, *p* = 0.02; FGF21: r = 0.31, *p* < 0.001). None of the biomarkers correlated with BMI, WHR, and HbA1c.

Patients treated with metformin compared to those without had lower excretion of FGF21 (median 1.47, IQR 0.83–3.09, and 2.24, 1.34–3.13 ng/mmol, respectively, *p* = 0.02). Patients managed with SGLT2 inhibitors demonstrated lower urinary CTGF (177, 122–325 and 275, 157–494 ng/mmol, *p* = 0.03). We did not find any differences in the excretion of the studied molecules depending on the treatment with sulfonylurea, GLP-1 analogues, DPP4 inhibitors, or insulin (all *p* > 0.05). However, in patients on insulin therapy, urinary BECN1 and Klotho demonstrated reverse relationships with daily insulin dose adjusted to body weight (BECN1: r = −0.33, *p* = 0.03; Klotho: r = −0.24, *p* = 0.02).

We found no associations of the excretion of the studied molecules with coronary artery disease, chronic heart failure, and myocardial infarction in medical history, as well as with smoking status.

### 3.3. Associations between the Urinary Biomarkers and CKD: Univariate Models

In ROC analysis, PTEN ≥ 1.10 ng/mmol was associated with both eGFR < 60 mL/min × 1.73 m^2^ or UACR ≥ 3.0 mg/mmol (Table 3). A reduced eGFR was also associated with high urinary CTGF and FGF21.

We found cut-off points for urinary BECN1 and FGF21 as factors associated with DF/NA, as well as for CTGF and FGF21 as factors associated with DF/EA.

### 3.4. Associations between the Urinary Biomarkers and CKD: Multivariate Models

After adjustment for age, sex, BMI, duration of diabetes, and HbA1c, urinary Klotho was significantly associated with UACR ≥ 3.0 mg/mmol in patients with T2D (OR = 0.96, 95% CI 0.93–0.9996 for each 10 ng/mmol of urinary Klotho, *p* = 0.048, Table 4).

In a similar model, urinary PTEN was associated with DRF/EA (OR = 6.23, 95% CI 1.43–27.1, *p* = 0.01, Table 5).

We failed to build any model for eGFR < 60 mL/min × 1.73 m^2^, as well as for DRF/NA and NRF/EA patterns of CKD, where urinary excretion of the assessed molecules was significant after adjustment for age, sex, BMI, diabetes duration, and HbA1c.

## 4. Discussion

In this study, we have assessed the urinary excretion of biomolecules related to cell cycle, proliferation, and autophagy (PTEN, BECN1, SIRT1, FGF21, Klotho, and CTGF) in patients with long-term T2D and different patterns of CKD. The results indicate that the studied regulators are differently related to albuminuria, renal function, and CKD phenotype. Specifically, urinary Klotho is associated with elevated albuminuria, while high excretion of FGF21, CTGF, and PTEN are associated with decreased renal function. We found elevated excretion of BECN1 in non-albuminuric T2D subjects, while Klotho and PTEN showed independent associations with albuminuric CKD patterns.

### 4.1. PTEN

We found high levels of urinary PTEN in patients with long-term T2D. The increased PTEN excretion adjusted to age, sex, BMI, diabetes duration, and HbA1c was associated with both declined eGFR and elevated UACR.

PTEN is a suppressor of the PI3K/AKT/mTOR signaling pathway and a crucial regulator of cell death, autophagy, and apoptosis [17,23,24]. In the human kidney, PTEN is expressed at a low or medium level in the tubular cells, and expressed at a low or zero level in the glomeruli [36]. PTEN is considered to be an antagonist of the transforming growth factor beta (TGF-β) signaling pathway [37]. The down-regulation of PTEN in podocytes was found in both clinical and experimental diabetes [38,39,40]. At the same time, it was demonstrated that high renal PTEN may increase expression of fibrogenic agents, such as TGF-β and CTGF, and promote epithelial-mesenchymal transition [41].

The exact mechanism of the increased urinary excretion of PTEN in diabetes is not clear. Assuming intracellular localization of PTEN, one can speculate that the elevated urinary excretion of this molecule could be a result of renal cell injury.

### 4.2. BECN1

We observed increased urinary excretion of BECN1 in patients with T2D, especially in those with non-albuminuric CKD.

BECN1 is an essential regulator of autophagy that signals the onset of the process [42]. A medium level of BECN1 expression in the renal glomeruli and tubules was described [36]. Similarly to PTEN, elevation of BECN1 excretion may be a consequence of an injury of the renal cells. Previously, we found a decreased glomerular expression of BECN1 in *db*/*db* mice, a model of T2D [43]. Accordingly, serum level of BECN1 was reported to be reduced in patients with T2D and CKD [44].

Further studies are needed to clarify changes in the synthesis of PTEN and BECN1 in diabetic kidneys and their role in increasing albuminuria and reducing renal function.

### 4.3. SIRT1

In this study, we revealed elevated urinary SIRT1 excretion in patients with T2D. However, we found no differences in SIRT1 excretion between groups of patients with different patterns of CKD.

In the kidney, SIRT1 is expressed in podocytes and proximal tubular epithelial cells [45]. It was demonstrated that renal SIRT1 inhibits cell apoptosis, inflammation, and fibrosis [45,46]. Therefore, activation of SIRT1 can be a protective mechanism in diabetic kidney disease [46]. The synthesis of SIRT1 in the diabetic kidney and its relationship with the urinary excretion of the molecule requires further research.

### 4.4. Klotho

The obtained results demonstrate the association between a decrease in the urinary excretion of Klotho and the elevation of albuminuria in patients with T2D.

Previously, decreased serum levels of Klotho were found in patients with T2D [47,48]. However, a study including 13,751 subjects from the National Health and Nutrition Examination Survey (NHANES) database revealed higher prevalence of T2D in people with serum levels of Klotho in the lower or upper quartiles (Q1, Q4) compared to those with Klotho in Q2 or Q3. This study also demonstrated lower eGFR in participants with serum levels of Klotho below 993.25 pg/mL [49]. The independent associations between serum levels of Klotho and eGFR as well as HbA1c were describes previously [50].

In the kidney, Klotho expresses in tubular cells predominantly [36]. The down-regulation of the renal Klotho was reported in kidney diseases [51,52,53]. In patients with CKD, a reduction of renal α-Klotho expression was associated with CKD progression [51,54]. Epigenetic modifications of Klotho gene promoters under the influence of serum uremic toxins can be responsible for the Klotho down-regulation [55].

Klotho protein is considered to have renoprotective activity as an antagonist of the TGF-β signaling pathway [37]. In animal models and cell cultures, Klotho deficiency enhanced renal inflammation and promoted fibrosis, while treatment with Klotho analogues mitigated these changes [56,57,58].

The associations between low serum and urinary Klotho and albuminuria in patients with T2D were noted in previous studies [48,59,60]. Some experimental data may help explain the mechanism of this relationship. It was demonstrated that albumin directly decreased Klotho mRNA and protein expression in cultured murine and human tubular cells [60]. This effect is mediated by endoplasmic reticulum stress and epigenetic modifications [60,61]. In albumiuria models, Klotho-ameliorated endoplasmic reticulum stress, decreased Ca^2+^ influx, and reduced cytoskeleton remodeling in podocytes [62,63].

Therefore, decreased urinary excretion of Klotho can be considered as a possible risk factor for albuminuric CKD in diabetes.

### 4.5. FGF21

We found associations between increased excretion of FGF21 and declined renal function.

FGF21 is expressed in the liver predominantly, and is secreted to blood; FGF21 expression was not detected in the kidney [36]. However, elevated serum FGF21 was found in patients with diabetic CKD [64,65].

In diabetes, FGF21 is commonly considered to be a protective factor due to its insulin-sensitizing and anti-inflammatory effects [66,67]. Recent studies have demonstrated antifibrotic activity of FGF21 in the kidney [68,69,70]. It was revealed that FGF21 needs to interact with Klotho in order to activate the FGF receptor [28]. Therefore, it can be assumed that a decrease in the renal production of Klotho in CKD contributes to FGF21 resistance and diminishes its protective effect.

### 4.6. CTGF

In our study, patients with declined renal function demonstrated higher urinary CTGF, regardless of the elevation of albuminuria.

Previously, elevated serum levels of CTGF were found in patients with diabetic CKD [71,72]. Under normal conditions, CTGF is expressed in the glomerular and tubular cells at a low level [36]. High glucose up-regulates CTGF in the proximal tubular cells [73], as well as in the mesangial and mesenchymal cells [74,75,76]. CTGF is considered to be a fibrogenic agent in diabetic kidney disease [77,78,79].

Therefore, the elevated urinary excretion of CTGF could be an indicator of renal fibrogenesis in T2D patients with decreased renal function.

### 4.7. Dysregulation of the PI3K/AKT/mTOR Pathway in the Pathogenesis of Diabetic CKD

A growing body of evidence indicates the role of the PI3K/AKT/mTOR signaling pathway in the pathogenesis of diabetic kidney disease [14,80,81]. Specifically, this signaling pathway is responsible for the activation of apoptosis, suppression of autophagy, and the epithelial-mesenchymal transformation of podocytes and tubular cells, proliferation of mesangial cells, and fibrogenesis. In agreement with these data, some novel pharmacological agents modulating the PI3K/AKT/mTOR pathway showed protective effects in diabetic kidney [82,83,84].

**Figure 2:**
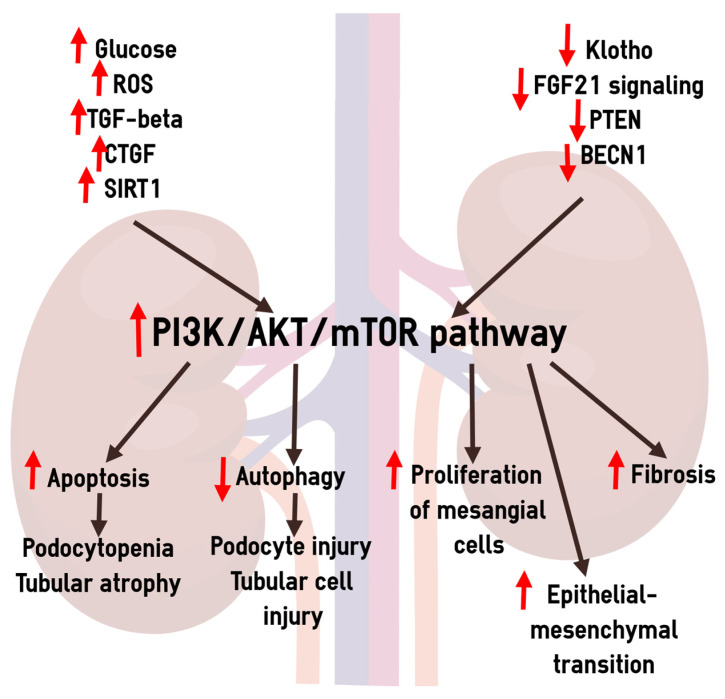
A schematic representation of the possible involvement of PTEN, BECN1, FGF21, Klotho, CTGF, and SIRT1 in the pathogenesis of diabetic kidney disease. PTEN, phosphatase and tensin homolog; BECN1, Beclin-1; FGF21, fibroblast growth factor 21; CTGF, connective tissue growth factor; SIRT1, sitruin 1; TGF-beta, transforming growth factor beta; ROS, reactive oxygen species; PI3K/AKT/mTOR, phosphatidylinositol 3-kinases/protein kinase B/mammalian target of the rapamycin. Red upward arrow indicates increased production of a molecule or activation of a process. Red downward arrow indicates decreased production of a molecule or suppression of a processes.

Hyperglycemia and oxidative stress are considered to be the primary factors activating the PI3K/AKT signaling in diabetes [13,14,85]. In addition, fibrogenic growth factors (TGF-β, CTGF) [14,16,32], elevated SIRT1 [34,35], decreased Klotho [26], PTEN [23,24], and BECN1 [86], as well as impaired FGF21 signaling [29,30,31], may contribute to the activation of the PI3K/AKT/mTOR pathway. A schematic representation of the role of PI3K/AKT/mTOR signaling in diabetic kidney disease is shown in Figure 2.

In this study, we tested hypothesis that molecules influencing the PI3K/AKT/mTOR signaling can be perspective biomarkers of albuminuric and/or non-albuminuric CKD in T2D. The results indicate that decreased urinary excretion of Klotho is related to increased albuminuria, FGF21, and CTGF are associated with declined renal function, PTEN is associated with both albuminuria and decreased eGFR, and BECN1 is associated with non-albuminuric CKD. It can be assumed that the pathogenetic significance of the studied regulators for increasing albuminuria and decreasing renal function is not the same.

### 4.8. Limitations of the Study and Future Remarks

This study is not without limitations. First of all, due to the variability of the eGFR and UACR, some patients may have been misclassified with CKD patterns. The limited sample size and cross-sectional design are other obvious limitations. Morphological verification of kidney pathology was not carried out.

However, to the best of our knowledge, this is the first study examining the excretion of biomolecules influencing the PI3K/AKT/mTOR signaling pathway in albuminuric and non-albuminuric CKD in T2D. Future translational studies are needed to clarify the pathogenetic role of the studied molecules in albuminuric and non-albuminuric diabetic CKD. The significance of the studied molecules as predictors of decreased renal function and increased albuminuria deserves testing in prospective study.

High glucose, reactive oxygen species (ROS), fibrogenic growth factors (TGF-β, CTGF), elevated SIRT1, decreased Klotho, PTEN, and BECN1, as well as impaired FGF21 signaling, can contribute to the activation of PI3K/AKT/mTOR. In its turn, activation of the PI3K/AKT/mTOR is important for apoptosis promoting, autophagy suppression, and epithelial-mesenchymal transition in the podocyte and tubular cells, proliferation of mesangial cells, and fibrosis.

## 5. Conclusions

In patients with long-term T2D, urinary PTEN, BECN1, FGF21, Klotho, and CTGF, the molecules that are involved in the regulation of cell cycle, proliferation, and autophagy, are associated differently with albuminuric and non-albuminuric patterns of CKD. Specifically, decreased urinary Klotho is related to increased albuminuria, FGF21 and CTGF are associated with declined renal function, PTEN is associated with both albuminuria and decreased eGFR, and BECN1 is associated with non-albuminuric CKD. The results provide further support for the role of PTEN, BECN1, FGF21, Klotho, and CTGF in the pathogenesis of diabetic kidney disease, and highlight the differences in the molecular pathways of albuminuric and non-albuminuric CKD in diabetes.

## Figures and Tables

**Table 1:** Clinical characteristics of patients with T2D depending on CKD status.

Parameter	Groups			
	NRF/NA	DRF/NA	NRF/EA	DRF/EA
N	35	35	35	35
Age, years	62 (56–66)	71 (65–75) ^^^	63 (58–68) ^##^	68 (61–71)
Sex (F/M), *n*	18/17	17/18	18/17	70/18
BMI, kg/m^2^	31.6 (29.0–35.7) *	31.4 (27.9–35.7) *	31.9 (28.7–35.7)	32.4 (27.5–35.7)
WHR	1.00 (0.94–1.07)	0.98 (0.93–1.08)	1.00 (0.95–1.06)	1.00 (0.98–1.10)
Smoking, *n* (%)	7 (20.0)	10 (28.6)	7 (20.0)	3 (8.6)
Duration of T2D, years	13 (10–15)	15 (11–20)	16 (12–21)	18 (12–23) ^
HbA1c, %	8.0 (7.3–9.3)	7.8 (6.8–8.8)	9.2 (8.0–10.9) ^^##^	8.6 (8.0–9.9)
Creatinine, μmol/L	81 (70–89)	113 (98–122) ^^^	84 (70–97)	114 (101–149) ^^^
eGFR, mL/min × 1.73 m^2^	81 (70–89)	113 (98–122) ^^^	84 (70–97)	114 (101–149) ^^^
CKD G1/G2/G3a/G3b/G4	7/28/0/0/0	0/0/27/8/0	7/28/0/0/0	0/0/22/11/2
UACR, mg/mmol	0.3 (0.2–0.4)	0.4 (0.3–0.6)	21.1 (7.3–57.7) ^^^^###^	43.6 (9.9–99.7) ^^^^###^
Arterial hypertension, *n* (%)	35 (100)	35 (100)	35 (100)	35 (100)
Coronary artery disease, *n* (%)	15 (42.9)	15 (42.9)	16 (45.7)	21 (60)
Chronic heart failure (NYHA class III or IV), *n* (%)	0 (0)	1 (2.9)	0 (0)	5 (14.3) ^*
Myocardial infarction in medical history, *n* (%)	6 (17.1)	7 (20)	9 (25.7)	14 (40) ^
Stroke in medical history, *n* (%)	2 (5.7)	4 (11.4)	4 (11.4)	4 (11.4)

NRF/NA, normal renal function/normal albuminuria group; DRF/NA, declined renal function/normal albuminuria group; NRF/EA, normal renal function/elevated albuminuria group; DRF/EA, declined renal function/elevated albuminuria group; BMI, body mass index; CKD, chronic kidney disease; HbA1c, hemoglobin A1c; T2D, type 2 diabetes; UACR, urinary albumin-to-creatinine ratio; WHR, waist-to-hip ratio. ^ *p* < 0.05, ^^^ *p* < 0.001 vs. NRF/NA, ^##^ *p* < 0.01, ^###^ *p* < 0.001 vs. DRF/NA, * *p* < 0.05 vs. NRF/EA.

**Table 2:** Treatment of patients with T2D and different CKD status.

Parameter	Groups			
	NRF/NA	DRF/NA	NRF/EA	DRF/EA
N	35	35	35	35
Antihyperglycemic agents				
Insulin, *n* (%)	21 (60)	22 (62.9)	24 (68.6)	29 (82.9)
Metformin, *n* (%)	34 (97.1)	24 (68.6)	31 (88.6)	16 (45.7) ^*
Sulfonylurea, *n* (%)	16 (45.7)	15 (42.9)	15 (42.9)	9 (25.7)
DPP4 inhibitors, *n* (%)	8 (22.9)	4 (11.4)	3 (8.6)	3 (8.6)
GLP-1 analogues, *n* (%)	1 (2.86)	1 (2.86)	0 (0)	0 (0)
SGLT2 inhibitors, *n* (%)	7 (20)	11 (31.4)	13 (37.1)	4 (11.4)
Antihypertensive agents				
ACE inhibitor/ ARB, *n* (%)	12/16 (34.3/45.7)	11/19 (31.4/54.3)	9/17 (25.7/48.6)	14/17 (40/48.6)
β-blockers, *n* (%)	15 (42.9)	24 (68.6)	25 (71.4)	24 (68.6)
CCB, *n* (%)	8 (22.9)	16 (45.7)	14 (40)	20 (57.1) ^
Diuretics, *n* (%)	16 (45.7)	19 (54.3)	14 (40)	24 (68.6)
Lipid-lowering agents				
Statins, *n* (%)	23 (65.7)	22 (62.9)	21 (60.0)	24 (68.6)

NRF/NA, normal renal function/normal albuminuria group; DRF/NA, declined renal function/normal albuminuria group; NRF/EA, normal renal function/elevated albuminuria group; DRF/EA, declined renal function/elevated albuminuria group; ACE, angiotensin-converting enzyme; ARB, angiotensin receptor blockers; BMI, body mass index; CCB, calcium channel blockers; CKD, chronic kidney disease; DPP4, dipeptidyl peptidase-4; GLP-1, glucagon-like peptide-1; SGLT2, sodium/glucose cotransporter 2; T2D, type 2 diabetes. ^ *p* < 0.05, * *p* < 0.05 vs. NRF/EA.

**Table 3:** Associations of urinary biomarkers with CKD in patients with T2D: The results of ROC-analysis.

Parameter	Cut-Off Point	AUC ± SE, 95% CI, *p*-Value	OR, 95% CI, *p*-Value	Se	Sp
eGFR < 60 mL/min × 1.73 m^2^					
FGF21	≥1.74 ng/mmol	0.6 ± 0.05 (0.504–0.69), *p* = 0.04	2.12 (1.08–4.16), *p* = 0.03	0.6	0.6
CTGF	≥230 ng/mmol	0.6 ± 0.05 (0.51–0.7), *p* = 0.04	2.13 (1.08–4.19), *p* = 0.03	0.57	0.57
PTEN	≥1.10 ng/mmol	0.64 ± 0.05 (0.55–0.73), *p* = 0.004	2.12 (1.08–4.17), *p* = 0.03	0.61	0.57
UACR ≥ 3.0 mg/mmol					
Klotho	≤24 pg/mmol	0.6 ± 0.05 (0.504–0.69), *p* = 0.047	1.58 (0.81–3.08), *p* = 0.18	0.56	0.56
PTEN	≥1.10 ng/mmol	0.61 ± 0.05 (0.51–0.7), *p* = 0.03	2.39 (1.21–4.72), *p* = 0.01	0.63	0.59
DRF/NA					
FGF21	≥1.49 ng/mmol	0.64 ± 0.07 (0.51–0.77), *p* = 0.046	2.86 (1.09–7.55), *p* = 0.03	0.63	0.63
BECN1	≥10 ng/mmol	0.73 ± 0.09 (0.55–0.92), *p* = 0.02	4.84 (1.09–21.6), *p* = 0.04	0.69	0.69
PTEN	≥1.03 ng/mmol	0.64 ± 0.07 (0.51–0.77), *p* = 0.048	1.78 (0.69–4.58), *p* = 0.23	0.57	0.57
DRF/EA					
FGF21	≥1.62 ng/mmol	0.66 ± 0.07 (0.53–0.79), *p* = 0.02	4.18 (1.54–11.4), *p* = 0.005	0.69	0.66
CTGF	≥251 ng/mmol	0.66 ± 0.07 (0.53–0.79), *p* = 0.02	2.86 (1.09–7.55), *p* = 0.03	0.63	0.63

CKD, chronic kidney disease; eGFR, estimated glomerular filtration rate; T2D, type 2 diabetes; UACR, urinary albumin-to-creatinine ratio; DRF/NA, declined renal function/normal albuminuria; DRF/EA, declined renal function/elevated albuminuria; PTEN, phosphatase and tensin homolog; BECN1, Beclin-1; FGF21, fibroblast growth factor 21; CTGF, connective tissue growth factor.

**Table 4:** The logistic regression model of UACR ≥3.0 mg/mmol.

Parameter	Crude OR, 95% CI, *p*-Value	Adjusted OR, 95% CI, *p*-Value
Urinary Klotho, 10 pg/mmol	0.97 (0.94–1.01), *p* = 0.1	0.96 (0.93–0.9996), *p* = 0.048
Age, years	1 (0.98–1.03), *p* = 0.91	0.99 (0.94–1.04), *p* = 0.61
Male sex	1.67 (1.31–2.14), *p* < 0.001	1.04 (0.71–1.52), *p* = 0.83
BMI, kg/m^2^	0.99 (0.94–1.05), *p* = 0.78	1 (0.92–1.09), *p* = 0.99
Duration of T2D, years	1.03 (0.99–1.06), *p* = 0.12	1.07 (1.01–1.13), *p* = 0.01
HbA1c, %	1.19 (1.05–1.35), *p* = 0.01	1.47 (1.16–1.85), *p* = 0.001

Parameters of the model: intercept −3.45; KS *p*-value < 0.001, AUC 0.74, Se 0.70, Sp 0.70 for L_P_ = 0.48. BMI, body mass index; CI, confidence interval; HbA1c, hemoglobin A1c; OR, odds ratio; T2D, type 2 diabetes; UACR, urinary albumin-to-creatinine ratio.

**Table 5:** The logistic regression model for DRF/EA pattern of CKD.

Parameter	Crude OR, 95% CI, *p*-Value	Adjusted OR, 95% CI, *p*-Value
Urinary PTEN, ng/mmol	6.33 (1.91–21.0), *p* = 0.003	6.23 (1.43–27.1), *p* = 0.01
Age, years	1.08 (1.03–1.13), *p* < 0.001	1.05 (0.97–1.14), *p* = 0.2
Male sex	1.31 (0.94–1.84), *p* = 0.11	1.08 (0.58–1.99), *p* = 0.82
BMI, kg/m^2^	1.0 (0.93–1.08), *p* = 0.96	0.93 (0.81–1.06), *p* = 0.27
Diabetes duration, years	1.07 (1.02–1.12), *p* = 0.003	1.12 (1.01–1.25), *p* = 0.04
HbA1c, %	1.1 (0.93–1.31), *p* = 0.26	1.33 (0.96–1.85), *p* = 0.09

Parameters of the model: intercept = −7.44; KS *p*-value < 0.001, AUC = 0.82, Se = 0.77, Sp = 0.77 for L_P_ = 0.49). BMI, body mass index; CI, confidence interval; HbA1c, hemoglobin A1c; OR, odds ratio; T2D, type 2 diabetes; DRF/EA, declined renal function/elevated albuminuria; PTEN, phosphatase and tensin homolog.

## Data Availability

The data presented in this study are available on request from the corresponding author.

## References

[1] Lv J.-C., Zhang L.-X. (2019). Prevalence and Disease Burden of Chronic Kidney Disease. Adv. Exp. Med. Biol..

[2] Magliano D.J., Boyko E.J., IDF Diabetes Atlas 10th Edition Scientific Committee (2021). IDF Diabetes Atlas.

[3] Zhang X.-X., Kong J., Yun K. (2020). Prevalence of Diabetic Nephropathy among Patients with Type 2 Diabetes Mellitus in China: A Meta-Analysis of Observational Studies. J. Diabetes Res..

[4] Ying M., Shao X., Qin H., Yin P., Lin Y., Wu J., Ren J., Zheng Y. (2023). Disease Burden and Epidemiological Trends of Chronic Kidney Disease at the Global, Regional, National Levels from 1990 to 2019. Nephron.

[5] Jayakumari C., Gomez R., Dipin S., Jayakumar R.V., Vijayakumar K., Sreenath R., Ajeesh T., Jabbar P.K., Das D.V., Gopi G.V. (2022). Prevalence of Normoalbuminuric Chronic Kidney Disease among Individuals with Type 2 Diabetes Mellitus from India. Indian J. Med. Res..

[6] Scilletta S., Di Marco M., Miano N., Filippello A., Di Mauro S., Scamporrino A., Musmeci M., Coppolino G., Di Giacomo Barbagallo F., Bosco G. (2023). Update on Diabetic Kidney Disease (DKD): Focus on Non-Albuminuric DKD and Cardiovascular Risk. Biomolecules.

[7] Klimontov V.V., Korbut A.I. (2019). Albuminuric and Non-Albuminuric Patterns of Chronic Kidney Disease in Type 2 Diabetes. Diabetes Metab. Syndr..

[8] Shi S., Ni L., Gao L., Wu X. (2022). Comparison of Nonalbuminuric and Albuminuric Diabetic Kidney Disease Among Patients with Type 2 Diabetes: A Systematic Review and Meta-Analysis. Front. Endocrinol..

[9] Dai Q., Chen N., Zeng L., Lin X.-J., Jiang F.-X., Zhuang X.-J., Lu Z.-Y. (2021). Clinical Features of and Risk Factors for Normoalbuminuric Diabetic Kidney Disease in Hospitalized Patients with Type 2 Diabetes Mellitus: A Retrospective Cross-Sectional Study. BMC Endocr. Disord..

[10] Tang C., Livingston M.J., Liu Z., Dong Z. (2020). Autophagy in Kidney Homeostasis and Disease. Nat. Rev. Nephrol..

[11] Sanz A.B., Sanchez-Niño M.D., Ramos A.M., Ortiz A. (2023). Regulated Cell Death Pathways in Kidney Disease. Nat. Rev. Nephrol..

[12] Mohandes S., Doke T., Hu H., Mukhi D., Dhillon P., Susztak K. (2023). Molecular Pathways That Drive Diabetic Kidney Disease. J. Clin. Investig..

[13] Klimontov V.V., Saik O.V., Korbut A.I. (2021). Glucose Variability: How Does It Work?. Int. J. Mol. Sci..

[14] Zhang Y., Jin D., Kang X., Zhou R., Sun Y., Lian F., Tong X. (2021). Signaling Pathways Involved in Diabetic Renal Fibrosis. Front. Cell Dev. Biol..

[15] Liu M., Ning X., Li R., Yang Z., Yang X., Sun S., Qian Q. (2017). Signalling Pathways Involved in Hypoxia-Induced Renal Fibrosis. J. Cell. Mol. Med..

[16] Zhang Y., Wang S., Liu S., Li C., Wang J. (2015). Role of Smad Signaling in Kidney Disease. Int. Urol. Nephrol..

[17] Xu J., Li Y., Kang M., Chang C., Wei H., Zhang C., Chen Y. (2023). Multiple Forms of Cell Death: A Focus on the PI3K/AKT Pathway. J. Cell. Physiol..

[18] Savova M.S., Mihaylova L.V., Tews D., Wabitsch M., Georgiev M.I. (2023). Targeting PI3K/AKT Signaling Pathway in Obesity. Biomed. Pharmacother..

[19] Verma K., Jaiswal R., Paliwal S., Dwivedi J., Sharma S. (2023). An Insight into PI3k/Akt Pathway and Associated Protein-Protein Interactions in Metabolic Syndrome: A Recent Update. J. Cell. Biochem..

[20] Maffei A., Lembo G., Carnevale D. (2018). PI3Kinases in Diabetes Mellitus and Its Related Complications. Int. J. Mol. Sci..

[21] Bilanges B., Posor Y., Vanhaesebroeck B. (2019). PI3K Isoforms in Cell Signalling and Vesicle Trafficking. Nat. Rev. Mol. Cell Biol..

[22] Gulluni F., De Santis M.C., Margaria J.P., Martini M., Hirsch E. (2019). Class II PI3K Functions in Cell Biology and Disease. Trends Cell Biol..

[23] Khokhar M., Roy D., Modi A., Agarwal R., Yadav D., Purohit P., Sharma P. (2020). Perspectives on the Role of PTEN in Diabetic Nephropathy: An Update. Crit. Rev. Clin. Lab. Sci..

[24] Kma L., Baruah T.J. (2022). The Interplay of ROS and the PI3K/Akt Pathway in Autophagy Regulation. Biotechnol. Appl. Biochem..

[25] Chen C.-Y., Chen J., He L., Stiles B.L. (2018). PTEN: Tumor Suppressor and Metabolic Regulator. Front. Endocrinol..

[26] Zhou H., Pu S., Zhou H., Guo Y. (2021). Klotho as Potential Autophagy Regulator and Therapeutic Target. Front. Pharmacol..

[27] Menon M.B., Dhamija S. (2018). Beclin 1 Phosphorylation—At the Center of Autophagy Regulation. Front. Cell Dev. Biol..

[28] Kuro-O M. (2019). The Klotho Proteins in Health and Disease. Nat. Rev. Nephrol..

[29] Liu Q., Huang J., Yan W., Liu Z., Liu S., Fang W. (2023). FGFR Families: Biological Functions and Therapeutic Interventions in Tumors. MedComm (2020).

[30] Mossahebi-Mohammadi M., Quan M., Zhang J.-S., Li X. (2020). FGF Signaling Pathway: A Key Regulator of Stem Cell Pluripotency. Front. Cell Dev. Biol..

[31] Agrawal S., Maity S., AlRaawi Z., Al-Ameer M., Kumar T.K.S. (2021). Targeting Drugs Against Fibroblast Growth Factor(s)-Induced Cell Signaling. Curr. Drug Targets.

[32] Cheng J.-C., Gao Y., Chen J., Meng Q., Fang L. (2023). EGF Promotes Human Trophoblast Cell Invasion by Downregulating CTGF Expression via PI3K/AKT Signaling. Reproduction.

[33] Wu Z., Zhou C., Yuan Q., Zhang D., Xie J., Zou S. (2021). CTGF Facilitates Cell-Cell Communication in Chondrocytes via PI3K/Akt Signalling Pathway. Cell Prolif..

[34] Pillai V.B., Sundaresan N.R., Gupta M.P. (2014). Regulation of Akt Signaling by Sirtuins: Its Implication in Cardiac Hypertrophy and Aging. Circ. Res..

[35] Meng F., Zhang Z., Chen C., Liu Y., Yuan D., Hei Z., Luo G. (2021). PI3K/AKT Activation Attenuates Acute Kidney Injury Following Liver Transplantation by Inducing FoxO3a Nuclear Export and Deacetylation. Life Sci..

[36] Uhlén M., Fagerberg L., Hallström B.M., Lindskog C., Oksvold P., Mardinoglu A., Sivertsson Å., Kampf C., Sjöstedt E., Asplund A. (2015). Proteomics. Tissue-Based Map of the Human Proteome. Science.

[37] Tang J., Gifford C.C., Samarakoon R., Higgins P.J. (2018). Deregulation of Negative Controls on TGF-Β1 Signaling in Tumor Progression. Cancers.

[38] Lin J., Shi Y., Peng H., Shen X., Thomas S., Wang Y., Truong L.D., Dryer S.E., Hu Z., Xu J. (2015). Loss of PTEN Promotes Podocyte Cytoskeletal Rearrangement, Aggravating Diabetic Nephropathy. J. Pathol..

[39] Wang H., Feng Z., Xie J., Wen F., Jv M., Liang T., Li J., Wang Y., Zuo Y., Li S. (2018). Podocyte-Specific Knockin of PTEN Protects Kidney from Hyperglycemia. Am. J. Physiol. Renal Physiol..

[40] Audzeyenka I., Rachubik P., Typiak M., Kulesza T., Kalkowska D., Rogacka D., Rychłowski M., Angielski S., Saleem M., Piwkowska A. (2022). PTEN-Induced Kinase 1 Deficiency Alters Albumin Permeability and Insulin Signaling in Podocytes. J. Mol. Med..

[41] Li Y., Hu Q., Li C., Liang K., Xiang Y., Hsiao H., Nguyen T.K., Park P.K., Egranov S.D., Ambati C.R. (2019). PTEN-Induced Partial Epithelial-Mesenchymal Transition Drives Diabetic Kidney Disease. J. Clin. Investig..

[42] Klionsky D.J., Abdel-Aziz A.K., Abdelfatah S., Abdellatif M., Abdoli A., Abel S., Abeliovich H., Abildgaard M.H., Abudu Y.P., Acevedo-Arozena A. (2021). Guidelines for the Use and Interpretation of Assays for Monitoring Autophagy (4th Edition)^1^. Autophagy.

[43] Korbut A.I., Taskaeva I.S., Bgatova N.P., Muraleva N.A., Orlov N.B., Dashkin M.V., Khotskina A.S., Zavyalov E.L., Konenkov V.I., Klein T. (2020). SGLT2 Inhibitor Empagliflozin and DPP4 Inhibitor Linagliptin Reactivate Glomerular Autophagy in Db/Db Mice, a Model of Type 2 Diabetes. Int. J. Mol. Sci..

[44] Naguib M., Rashed L.A. (2018). Serum Level of the Autophagy Biomarker Beclin-1 in Patients with Diabetic Kidney Disease. Diabetes Res. Clin. Pract..

[45] Morigi M., Perico L., Benigni A. (2018). Sirtuins in Renal Health and Disease. J. Am. Soc. Nephrol..

[46] Qi W., Hu C., Zhao D., Li X. (2022). SIRT1-SIRT7 in Diabetic Kidney Disease: Biological Functions and Molecular Mechanisms. Front. Endocrinol..

[47] Timoshchenko O.V., Stakhneva E.M., Ragino Y.I., Nikitin Y.P. (2021). Klotho Protein in Men with Type 2 Diabetes Mellitus Blood and Its Association with Cardiometabolic Risk Factors. Sib. Nauchnyj Med. Zhurnal.

[48] Nie F., Wu D., Du H., Yang X., Yang M., Pang X., Xu Y. (2017). Serum Klotho Protein Levels and Their Correlations with the Progression of Type 2 Diabetes Mellitus. J. Diabetes Complicat..

[49] Wang K., Mao Y., Lu M., Liu X., Sun Y., Li Z., Li Y., Ding Y., Zhang J., Hong J. (2022). Association between Serum Klotho Levels and the Prevalence of Diabetes among Adults in the United States. Front. Endocrinol..

[50] Typiak M., Kulesza T., Rachubik P., Rogacka D., Audzeyenka I., Angielski S., Saleem M.A., Piwkowska A. (2021). Role of Klotho in Hyperglycemia: Its Levels and Effects on Fibroblast Growth Factor Receptors, Glycolysis, and Glomerular Filtration. Int. J. Mol. Sci..

[51] Martín-Vírgala J., Fernández-Villabrille S., Martín-Carro B., Tamargo-Gómez I., Navarro-González J.F., Mora-Fernández C., Calleros L., Astudillo-Cortés E., Avello-Llano N., Mariño G. (2023). Serum and Urinary Soluble α-Klotho as Markers of Kidney and Vascular Impairment. Nutrients.

[52] Xia J., Cao W. (2021). Epigenetic Modifications of Klotho Expression in Kidney Diseases. J. Mol. Med..

[53] Kale A., Sankrityayan H., Anders H.-J., Gaikwad A.B. (2021). Epigenetic and Non-Epigenetic Regulation of Klotho in Kidney Disease. Life Sci..

[54] Sakan H., Nakatani K., Asai O., Imura A., Tanaka T., Yoshimoto S., Iwamoto N., Kurumatani N., Iwano M., Nabeshima Y.-I. (2014). Reduced Renal α-Klotho Expression in CKD Patients and Its Effect on Renal Phosphate Handling and Vitamin D Metabolism. PLoS ONE.

[55] Portales-Castillo I., Simic P. (2022). PTH, FGF-23, Klotho and Vitamin D as Regulators of Calcium and Phosphorus: Genetics, Epigenetics and Beyond. Front. Endocrinol..

[56] Yuan Q., Ren Q., Li L., Tan H., Lu M., Tian Y., Huang L., Zhao B., Fu H., Hou F.F. (2022). A Klotho-Derived Peptide Protects against Kidney Fibrosis by Targeting TGF-β Signaling. Nat. Commun..

[57] Fu Y., Cao J., Wei X., Ge Y., Su Z., Yu D. (2023). Klotho Alleviates Contrast-Induced Acute Kidney Injury by Suppressing Oxidative Stress, Inflammation, and NF-KappaB/NLRP3-Mediated Pyroptosis. Int. Immunopharmacol..

[58] Zhao Y., Banerjee S., Dey N., LeJeune W.S., Sarkar P.S., Brobey R., Rosenblatt K.P., Tilton R.G., Choudhary S. (2011). Klotho Depletion Contributes to Increased Inflammation in Kidney of the Db/Db Mouse Model of Diabetes via RelA (Serine)^536^ Phosphorylation. Diabetes.

[59] Chang K., Li Y., Qin Z., Zhang Z., Wang L., Yang Q., Su B. (2023). Association between Serum Soluble α-Klotho and Urinary Albumin Excretion in Middle-Aged and Older US Adults: NHANES 2007–2016. J. Clin. Med..

[60] Fernandez-Fernandez B., Izquierdo M.C., Valiño-Rivas L., Nastou D., Sanz A.B., Ortiz A., Sanchez-Niño M.D. (2018). Albumin Downregulates Klotho in Tubular Cells. Nephrol. Dial. Transplant..

[61] Delitsikou V., Jarad G., Rajaram R.D., Ino F., Rutkowski J.M., Chen C.-D., Santos C.X.C., Scherer P.E., Abraham C.R., Shah A.M. (2020). Klotho Regulation by Albuminuria Is Dependent on ATF3 and Endoplasmic Reticulum Stress. FASEB J..

[62] Charrin E., Dabaghie D., Sen I., Unnersjö-Jess D., Möller-Hackbarth K., Burmakin M., Mencke R., Zambrano S., Patrakka J., Olauson H. (2023). Soluble Klotho Protects against Glomerular Injury through Regulation of ER Stress Response. Commun. Biol..

[63] Kim J.-H., Xie J., Hwang K.-H., Wu Y.-L., Oliver N., Eom M., Park K.-S., Barrezueta N., Kong I.-D., Fracasso R.P. (2017). Klotho May Ameliorate Proteinuria by Targeting TRPC6 Channels in Podocytes. J. Am. Soc. Nephrol..

[64] Suassuna P.G.d.A., de Paula R.B., Sanders-Pinheiro H., Moe O.W., Hu M.-C. (2019). Fibroblast Growth Factor 21 in Chronic Kidney Disease. J. Nephrol..

[65] Catalina M.O.-S., Redondo P.C., Granados M.P., Cantonero C., Sanchez-Collado J., Albarran L., Lopez J.J. (2019). New Insights into Adipokines as Potential Biomarkers for Type-2 Diabetes Mellitus. Curr. Med. Chem..

[66] Fisher F.M., Maratos-Flier E. (2016). Understanding the Physiology of FGF21. Annu. Rev. Physiol..

[67] Flippo K.H., Potthoff M.J. (2021). Metabolic Messengers: FGF21. Nat. Metab..

[68] Zhang C., Shao M., Yang H., Chen L., Yu L., Cong W., Tian H., Zhang F., Cheng P., Jin L. (2013). Attenuation of Hyperlipidemia- and Diabetes-Induced Early-Stage Apoptosis and Late-Stage Renal Dysfunction via Administration of Fibroblast Growth Factor-21 Is Associated with Suppression of Renal Inflammation. PLoS ONE.

[69] Lin S., Yu L., Ni Y., He L., Weng X., Lu X., Zhang C. (2020). Fibroblast Growth Factor 21 Attenuates Diabetes-Induced Renal Fibrosis by Negatively Regulating TGF-β-P53-Smad2/3-Mediated Epithelial-to-Mesenchymal Transition via Activation of AKT. Diabetes Metab. J..

[70] Weng W., Ge T., Wang Y., He L., Liu T., Wang W., Zheng Z., Yu L., Zhang C., Lu X. (2020). Therapeutic Effects of Fibroblast Growth Factor-21 on Diabetic Nephropathy and the Possible Mechanism in Type 1 Diabetes Mellitus Mice. Diabetes Metab. J..

[71] Dendooven A., Gerritsen K.G., Nguyen T.Q., Kok R.J., Goldschmeding R. (2011). Connective Tissue Growth Factor (CTGF/CCN2) ELISA: A Novel Tool for Monitoring Fibrosis. Biomarkers.

[72] Zhang H., Cai X., Yi B., Huang J., Wang J., Sun J. (2014). Correlation of CTGF Gene Promoter Methylation with CTGF Expression in Type 2 Diabetes Mellitus with or without Nephropathy. Mol. Med. Rep..

[73] Tang S.C.W., Leung J.C.K., Lai K.N. (2011). Diabetic Tubulopathy: An Emerging Entity. Contrib. Nephrol..

[74] Li X., Liu W., Wang Q., Liu P., Deng Y., Lan T., Zhang X., Qiu B., Ning H., Huang H. (2009). Emodin Suppresses Cell Proliferation and Fibronectin Expression via p38MAPK Pathway in Rat Mesangial Cells Cultured under High Glucose. Mol. Cell. Endocrinol..

[75] Du J., Wang L., Liu X., Zhou H., Fan Q., Luo J., Yao L., Wang J., Feng J., Ma J. (2010). Janus Kinase 2/Signal Transducers and Activators of Transcription Signal Inhibition Regulates Protective Effects of Probucol on Mesangial Cells Treated with High Glucose. Biol. Pharm. Bull..

[76] Yi B., Zhang H., Zhou H., Cai X., Sun J., Liu Y. (2011). High glucose induce the demethylation of CTGF promoter and gene expression. Xi Bao Yu Fen Zi Mian Yi Xue Za Zhi.

[77] Gifford C.C., Tang J., Costello A., Khakoo N.S., Nguyen T.Q., Goldschmeding R., Higgins P.J., Samarakoon R. (2021). Negative Regulators of TGF-Β1 Signaling in Renal Fibrosis; Pathological Mechanisms and Novel Therapeutic Opportunities. Clin. Sci..

[78] Lopes T.G., de Souza M.L., da Silva V.D., Dos Santos M., da Silva W.I.C., Itaquy T.P., Garbin H.I., Veronese F.V. (2019). Markers of Renal Fibrosis: How Do They Correlate with Podocyte Damage in Glomerular Diseases?. PLoS ONE.

[79] Chen X.-M., Qi W., Pollock C.A. (2009). CTGF and Chronic Kidney Fibrosis. Front. Biosci. (Schol. Ed.).

[80] Zdychová J., Kazdová L., Pelikanová T., Lindsley J.N., Anderson S., Komers R. (2008). Renal Activity of Akt Kinase in Obese Zucker Rats. Exp. Biol. Med..

[81] Horita S., Nakamura M., Suzuki M., Satoh N., Suzuki A., Seki G. (2016). Selective Insulin Resistance in the Kidney. BioMed Res. Int..

[82] Zhang Y., Wang Y., Luo M., Xu F., Lu Y., Zhou X., Cui W., Miao L. (2019). Elabela Protects against Podocyte Injury in Mice with Streptozocin-Induced Diabetes by Associating with the PI3K/Akt/mTOR Pathway. Peptides.

[83] Gao C., Fei X., Wang M., Chen Q., Zhao N. (2022). Cardamomin Protects from Diabetes-Induced Kidney Damage through Modulating PI3K/AKT and JAK/STAT Signaling Pathways in Rats. Int. Immunopharmacol..

[84] Dong R., Zhang X., Liu Y., Zhao T., Sun Z., Liu P., Xiang Q., Xiong J., Du X., Yang X. (2023). Rutin Alleviates EndMT by Restoring Autophagy through Inhibiting HDAC1 via PI3K/AKT/mTOR Pathway in Diabetic Kidney Disease. Phytomedicine.

[85] Ma X., Ma J., Leng T., Yuan Z., Hu T., Liu Q., Shen T. (2023). Advances in Oxidative Stress in Pathogenesis of Diabetic Kidney Disease and Efficacy of TCM Intervention. Ren. Fail..

[86] Jian M., Yunjia Z., Zhiying D., Yanduo J., Guocheng J. (2019). Interleukin 7 Receptor Activates PI3K/Akt/mTOR Signaling Pathway via Downregulation of Beclin-1 in Lung Cancer. Mol. Carcinog..

